# Stage-specific miR-188-5p and ER stress markers reflect tumor aggressiveness in prostate cancer

**DOI:** 10.3389/fonc.2026.1886215

**Published:** 2026-10-16

**Authors:** Hasan Riza Aydin, Ibrahim Sibal, Orhan Semerci, Hamit Zafer Aksoy, Fevzi Bedir, Volkan Enes Çelik, Sema Misir, Serap Ozer Yaman

**Affiliations:** 1 Department of Urology, Faculty of Medicine, Trabzon University, Trabzon Kanuni Training and Research Hospital, Trabzon, Türkiye; 2 Department of Pathology, Trabzon Kanuni Training and Research Hospital, Trabzon, Türkiye; 3 Department of Urology, Erzurum Faculty of Medicine, Health Sciences University, Erzurum City Hospital, Erzurum, Türkiye; 4 Department of Biochemistry, Faculty of Pharmacy, Sivas Cumhuriyet University, Sivas, Türkiye; 5 Department of Medical Biochemistry, Faculty of Medicine, Trabzon University, Trabzon Kanuni Training and Research Hospital, Trabzon, Türkiye

**Keywords:** ER stress, GRP78/CHOP axis, microRNA, prostate cancer, tumor aggressiveness

## Abstract

**Background:**

Reliable biomarkers are required to improve the detection and risk stratification of prostate cancer (PCa), particularly for identifying aggressive disease. MicroRNAs (miRNAs) have emerged as promising candidates; however, their integration with endoplasmic reticulum (ER) stress pathways and their clinical relevance remain incompletely defined.

**Methods:**

This study included 38 patients with histologically confirmed PCa and 20 individuals with benign biopsy findings. Patients were stratified into low–intermediate and high-risk groups based on clinical stage, Gleason score, and PSA levels. Expression levels of miR-188-5p, GRP78, and CHOP were quantified in prostate biopsy specimens using quantitative real-time PCR, and ELISA measured serum GRP78 and CHOP levels. In parallel, bioinformatic analyses were performed to predict candidate miR-188-5p interactions and to examine ER stress-related expression patterns using publicly available resources, including miRWalk, Sfold/STarMir, and GEPIA3 (TCGA/GTEx datasets). Associations between molecular markers and clinicopathological parameters were evaluated, and diagnostic performance was assessed using receiver operating characteristic (ROC) analysis and exploratory univariate logistic regression.

**Results:**

Bioinformatic analyses identified HSPA5 (GRP78) and DDIT3 (CHOP) as candidate miR-188-5p targets with predicted binding sites. External dataset analysis demonstrated higher HSPA5 and lower DDIT3 expression in prostate adenocarcinoma than in normal tissues. These computational findings indicate predicted interactions and do not establish direct binding or regulation. Clinically, miR-188-5p and GRP78 expression levels were significantly elevated in PCa patients compared with controls and increased progressively with tumor aggressiveness (p<0.001), whereas CHOP expression decreased (p<0.001). miR-188-5p was positively correlated with GRP78 and inversely correlated with CHOP. Among the evaluated markers, miR-188-5p showed the highest diagnostic discrimination in this cohort, and the exploratory composite biomarker model achieved an AUC of 0.966. Elevated miR-188-5p and GRP78 levels were also associated with adverse pathological features, including higher tumor grade and cribriform pattern; however, these findings require independent validation.

**Conclusion:**

Stage-associated alterations in miR-188-5p and the ER stress markers GRP78 and CHOP were associated with prostate cancer aggressiveness. The integrated computational and clinical findings support a potential expression-level relationship that may inform future biomarker studies, but they do not demonstrate direct miRNA–target binding or causal regulation.

## 1 Introduction

Prostate cancer (PCa) remains one of the most frequently diagnosed malignancies among men worldwide and represents a major cause of cancer-related mortality (1). Despite advances in prostate-specific antigen (PSA) screening, multiparametric imaging, and targeted therapies, a substantial proportion of patients progress to aggressive or castration resistant disease (2). The biological heterogeneity of PCa underscores the urgent need for reliable molecular biomarkers to improve risk stratification and guide therapeutic decision-making (3).

Alongside androgen receptor (AR)-mediated oncogenic signaling, increasing research emphasizes the essential function of cellular stress adaptation pathways in the advancement of prostate tumors. Endoplasmic reticulum (ER) stress and the unfolded protein response (UPR) have become crucial regulators of tumor cell survival, immune modulation, and therapy resistance (4, 5). Transient UPR activation restores cellular homeostasis, but long-term ER stress in cancer cells leads to adaptive survival signaling, immune evasion, and the ability to spread to other parts of the body (metastasis). Glucose-Regulated Protein 78 (GRP78, HSPA5) is a key chaperone in the endoplasmic reticulum (ER) that controls the activation of the unfolded protein response (UPR). It has also been linked to the progression of prostate cancer and resistance to castration. Increased GRP78 expression is linked to a worse prognosis and a more aggressive tumor (6, 7). Likewise, C/EBP homologous protein (CHOP, DDIT3), which has long been thought of as a pro-apoptotic UPR mediator, acts differently in different types of cancer and may help tumors adapt when ER stress is high. Research studies have shown that changing the levels of ER stress directly affects what happens to prostate cancer cells (4).

MicroRNAs (miRNAs) are small non-coding RNAs (20–23 nucleotides) that control gene expression after transcription and are important for both oncogenic and tumor-suppressive pathways. Dysregulation of particular miRNAs plays a role in the development, progression, metastasis, and resistance to therapy of prostate cancer (8). Among these, miR-188-5p has been identified as a tumor-suppressive miRNA in various malignancies, including prostate cancer, where it is linked to the inhibition of proliferation and metastasis (9). Nonetheless, its interaction with ER stress–related signaling in prostate cancer specimens is still inadequately defined. Studies indicate that specific miRNAs may modulate ER stress-related genes and affect apoptotic or adaptive survival pathways in prostate cancer cells. However, the precise correlation between miR-188-5p expression and established ER stress indicators like GRP78 and CHOP in human prostate cancer tissues has not been thoroughly investigated.

The coordinated assessment of miR-188-5p, GRP78, and CHOP may reveal clinically relevant expression patterns associated with prostate cancer aggressiveness. We hypothesized that ER stress-marker expression varies with tumor aggressiveness and that miR-188-5p expression is associated with GRP78 and/or CHOP levels. Accordingly, this descriptive, hypothesis-generating study examined the expression profiles of miR-188-5p, GRP78, and CHOP in prostate biopsy specimens across clinical risk categories, evaluated their clinicopathological associations, and explored predicted miRNA–target interactions using bioinformatic tools. Direct binding and causal regulatory effects were not tested.

## 2 Materials and methods

### 2.1 Patient selection

The study included a total of 38 prostate cancer patients, categorized as low–intermediate risk and high risk, along with a control group of 20 individuals. All participants were recruited from the Urology Clinic at Trabzon University and the Trabzon Kanuni Training and Research Hospital. Transrectal ultrasound (TRUS)- guided biopsy samples from all subjects were sent for pathological examination to confirm the diagnosis. The exclusion criteria for both patient and control groups were as follows: individuals under 18 years of age, patients who had received androgen deprivation therapy (ADT) or radiotherapy for prostate cancer, and those with a history of any concurrent malignancies. Inclusion criteria included men aged 40 years and older who had undergone a prostate biopsy, with biopsy indication based on suspicious digital rectal examination (DRE) findings or prostate-specific antigen (PSA) levels exceeding age-specific reference thresholds.

Participants were divided into three groups according to their biopsy results and clinical risk classification:

Control group: Individuals with biopsy results negative for malignancy, as well as high-grade prostatic intraepithelial neoplasia (HGPIN) or atypical small acinar proliferation (ASAP).

Low–intermediate risk prostate cancer group: Patients with clinical stage T1–T2, Gleason score ≤6 (low risk) or Gleason score 7 (intermediate risk), and PSA levels below 20 ng/mL.

High-risk prostate cancer group: Patients fulfilling at least one of the following: clinical stage T3 or 4, Gleason score above 7, or PSA exceeding 20 ng/mL.

All clinical and pathological data about the participants, including age, multiparametric magnetic resonance imaging (mp-MRI) findings, total prostate-specific antigen (tPSA) and free PSA (fPSA) levels, PSA density (PSAD), prostate volume, biopsy-derived Gleason score, and ISUP grade group, as well as additional pathological parameters and laboratory analyses, were retrospectively retrieved from hospital records. Written informed consent was obtained from all individuals included in the study prior to participation. The study was approved by the Non-Interventional Scientific Research Ethics Committee of Trabzon University Faculty of Medicine (Approval No: 2025/01; October 6, 2025) and adhered to the principles outlined in the Declaration of Helsinki.

### 2.2 miRNA, RNA extraction and quantitative real-time PCR

Total RNA was extracted from prostate biopsy specimens using a QIAGEN RNA extraction kit (QIAGEN, Cat. No. 217084, Hilden, Germany) according to the manufacturer’s instructions. Biopsy specimens allocated for molecular analysis were stored at −80 °C until RNA extraction. Complementary DNA was synthesized from the extracted RNA. For mRNA analysis, reverse transcription was performed using the OneScript Plus cDNA Synthesis Kit (Applied Biological Materials Inc., Cat. No. G236, Richmond, BC, Canada), following the manufacturer’s protocol. qPCR was performed on the LightCycler 480 system (Roche, Basel, Switzerland).

Thermal cycling comprised pre-incubation at 95 °C for 15 min, followed by 40 amplification cycles at 94 °C for 15 s, 60 °C for 30 s, and 72 °C for 30 s; melting-curve and cooling steps were then performed according to the instrument protocol. Expression levels of miR-188-5p, GRP78, and CHOP were determined by RT-qPCR. U6 small nuclear RNA was used as the endogenous reference for miR-188-5p, whereas GAPDH was used for GRP78 and CHOP. All reactions were performed in technical triplicate for each biological sample, and the mean cycle-threshold value was used for relative-expression analysis with the 2^−ΔΔCt method.

### 2.3 Measurement of serum GRP78 and CHOP levels by ELISA

Serum GRP78 and CHOP concentrations were measured using commercially available human sandwich enzyme-linked immunosorbent assay (ELISA) kits (GRP78: BT Laboratory, Cat. No. E3624Hu; CHOP/DDIT3: BT Laboratory, Cat. No. E4838Hu; Shanghai, China), according to the manufacturer’s instructions. Serum samples were stored at −80 °C until analysis and were allowed to reach room temperature before testing. Repeated freeze–thaw cycles were avoided. Standards and serum samples were analyzed in duplicate. Samples, standards, and blanks were added to the appropriate wells of the antibody-precoated microplates. Biotinylated detection antibody and streptavidin–horseradish peroxidase conjugate were subsequently added according to the manufacturer’s protocol. Following incubation, the plates were washed to remove unbound components. Substrate solutions were then added, and the enzymatic reaction was terminated using the supplied stop solution. Optical density was measured at 450 nm using a microplate reader (VERSA max, Molecular Devices, San Jose, CA, USA). A standard curve was generated separately for each assay plate. Serum concentrations were interpolated from the corresponding standard curves, and the mean value of duplicate measurements was used in the statistical analyses. Where applicable, sample dilution factors were included in the final calculations. Results were expressed as ng/mL. The detection ranges were 0.5–100 ng/mL for GRP78 and 0.2–60 ng/mL for CHOP, with reported analytical sensitivities of 0.23 and 0.097 ng/mL, respectively. (ELISA) kits. The results were expressed in ng/mL.

### 2.4 Bioinformatics analyses

The miRWalk database (http://mirwalk.umm.uni-heidelberg.de/) was used to identify predicted miR-188-5p binding sites across the 3′ untranslated region (3′UTR), coding sequence, and 5′ untranslated region of candidate genes. miRWalk integrates TarPmiR-based prediction with other resources and was used here as an exploratory screening tool. Predicted interactions were not considered experimentally validated unless supported by direct functional evidence; therefore, HSPA5 and DDIT3 were treated as candidate targets rather than confirmed downstream targets (10).

Software for Statistical Folding of Nucleic Acids and Analysis of Regulatory RNAs (Sfold) was used to identify potential miRNA-binding sites in the targeted genes, and the “STarMir” module in the Sfold database was selected (11).

The interactive web-based platform Gene Expression Profiling Interactive Analysis 3 (GEPIA3) offers a variety of visualization and analytical tools for gene expression analysis, based on data from The Cancer Genome Atlas (TCGA) and the Genotype-Tissue Expression (GTEx) project. Using the GEPIA gene expression profiling database (https://gepia3.bioinfoliu.com/Expression_network/, accessed on 3 February 2026), differences in the expression levels of selected genes between PRAD samples and normal tissues were evaluated. To ensure data integrity and intergroup comparability in gene expression analyses, all RNA-seq datasets were normalized by converting them to a log2(TPM + 1) (Transcripts Per Million) scale prior to analysis. The Fold Change (Log2FC) value, used to determine expression differences between groups, was calculated as the difference between the median values ​​of tumor and normal samples (log2FC = median(Tumor) - median(Normal)) (12).

### 2.5 Statistical analysis

Statistical analyses were carried out using IBM SPSS Statistics software (Version 23.0; IBM Corp., Armonk, NY, USA). The distribution of continuous variables was assessed with the Shapiro–Wilk test. As the data did not meet the assumptions of normality, nonparametric methods were employed. Comparisons among three groups for continuous variables were performed using the Kruskal–Wallis test, followed by Mann–Whitney U tests with Bonferroni adjustment for pairwise comparisons. Categorical variables were analyzed using the chi-square test or Fisher–Freeman–Halton/Fisher’s exact test, as appropriate. Associations among molecular biomarkers were examined using Spearman’s rank correlation. Diagnostic performance was evaluated using receiver operating characteristic curves and area under the curve (AUC). Because the modest sample size (38 PCa cases and 20 controls) produced unstable estimates in multivariable modeling, inferential regression results were restricted to exploratory univariate logistic regression with odds ratios and 95% confidence intervals. No claim of independent prediction was made. Age-adjusted multivariable estimates were not reported because the available sample did not support a stable adjusted model. A p-value <0.05 was considered statistically significant.

## 3 Results

Participants were stratified into high-risk prostate cancer (n = 18), low-to-intermediate-risk prostate cancer (n = 20), and benign control (n = 20) groups. Baseline demographic and clinicopathological characteristics are summarized in **Table 1**. Age differed across the three groups (overall p = 0.043); specifically, participants in the high-risk group were older than controls after Bonferroni adjustment (p = 0.033), whereas the other pairwise comparisons were not significant. Gleason score, ISUP grade, clinical stage, and adverse pathological findings were associated with tumor-risk category. Because age differed between groups and a stable age-adjusted model could not be fitted in this small cohort, residual confounding by age cannot be excluded.

**Table 1:** Demographic and clinicopathological characteristics of the study groups.

Variable	Control (n=20)	Low–intermediate PCa (n=20)	High-risk PCa (n=18)	p-value	Control vs low–intermediate	Control vs high-risk	Low–intermediate vs high-risk
Age (years)	65.0 ± 6.3	66.1 ± 7.2	70.7 ± 7.0	0.043	1.000	0.033	0.268
PSA (ng/mL)	6.58 ± 3.98	7.73 ± 5.37	28.13 ± 17.45	0.001	1.000	0.001	0.001
Prostate volume (mL)	79.5 ± 68.8	52.5 ± 19.5	51.0 ± 26.9	0.095	0.257	0.166	1.000
PSA density	0.105 ± 0.058	0.143 ± 0.068	0.686 ± 0.522	0.023	0.216	0.003	0.012
MRI PI-RADS score, median (IQR)	3.0 (3.0–4.0)	3.5 (3.0–4.0)	4.0 (4.0–5.0)	0.015	1.000	0.019	0.016
Any comorbidity, present	13/20 (65.0%)	13/20 (65.0%)	14/18 (77.8%)	0.639	1.000	1.000	1.000
Clinical stage, n (%)				0.001			
cT1	17 (85.0%)	16 (80.0%)	3 (16.7%)				
cT2a	1 (5.0%)	3 (15.0%)	6 (33.3%)				
cT2b	2 (10.0%)	1 (5.0%)	7 (38.9%)				
cT2c	0 (0.0%)	0 (0.0%)	2 (11.1%)				
cT3	0 (0.0%)	0 (0.0%)	0 (0.0%)				
Biopsy histopathology/Gleason–ISUP distribution, n (%)							
Benign/no malignancy	20 (100.0%)	0 (0.0%)	0 (0.0%)	0.001			
Gleason 6 (3 + 3), ISUP grade 1	0 (0.0%)	15 (75.0%)	0 (0.0%)	0.001	0.001	1.000	0.001
Gleason 7 (3 + 4), ISUP grade 2	0 (0.0%)	2 (10.0%)	6 (33.3%)	0.001	0.001	0.001	0.011
Gleason 7 (4 + 3), ISUP grade 3	0 (0.0%)	3 (15.0%)	5 (27.8%)	0.001	0.001	0.001	1.000
Gleason 8 (4 + 4), ISUP grade 4	0 (0.0%)	0 (0.0%)	5 (27.8%)	0.001	1.000	0.001	0.001
Gleason ≥9, ISUP grade 5	0 (0.0%)	0 (0.0%)	2 (11.1%)	0.001	1.000	0.001	0.001
Lymphovascular invasion	0/20 (0.0%)	0/20 (0.0%)	1/18 (5.6%)	0.310	1.000	1.000	1.000
Perineural invasion	0/20 (0.0%)	7/20 (35.0%)	11/18 (61.1%)	0.011	0.025	0.001	0.578
Cribriform pattern	0/20 (0.0%)	2/20 (10.0%)	12/18 (66.7%)	0.001	1.000	0.001	0.002

Data are mean ± SD, median (IQR), or n/N (%). Overall tests were Kruskal–Wallis or Fisher–Freeman–Halton; pairwise tests were Mann–Whitney U or Fisher’s exact tests with Bonferroni adjustment.

PSA-density values were obtained from the completed dataset. The Gleason/ISUP distribution P value compares the two prostate-cancer groups only.

Extraprostatic extension was not evaluated because the study used needle-biopsy specimens; this feature is generally assessed reliably in radical prostatectomy specimens and was therefore not included in the statistical table.

IQR, interquartile range; ISUP, International Society of Urological Pathology; PCa, prostate cancer; PI-RADS, Prostate Imaging Reporting and Data System; PSA, prostate-specific antigen.

The high-grade prostate cancer group had much higher serum PSA levels than the control and low-to-intermediate risk groups (p=0.0001). The prostate volume, on the other hand, did not differ significantly between the groups (p = 0.095).

PSA density (PSAD) was markedly increased in the high-grade group compared with controls (p = 0.003), indicating a correlation with tumor burden.

Analysis of molecular markers revealed a significant increase in miR-188-5p expression in the low- and intermediate-grade groups compared to controls, with expression levels peaking in the high-grade group (p = 0.001). The expression of GRP78 increased similarly as the severity of the disease increases, with the highest levels seen in high-grade prostate cancer (p=0.001). Conversely, CHOP expression decreased with increasing tumor aggressiveness (p = 0.001).

Serum analyses demonstrated consistent findings; serum GRP78 levels increased significantly with disease progression, whereas serum CHOP levels decreased significantly across the groups (p = 0.001 for both) (**Table 2**).

**Table 2:** Tissue and serum molecular parameters in the study groups.

Parameter	Control (n=20)	Low-Intermediate PCa (n=20)	High-Grade PCa (n=18)	p-value	Control vs low–intermediate	Control vs high-risk	Low–intermediate vs high-risk
miR-188-5p (fold)	2.19 ± 0.61	3.74 ± 0.69	7.11 ± 1.37	0.001	0.001	0.001	0.001
GRP78 (fold)	2.30 ± 0.71	2.98 ± 0.68	4.20 ± 0.73	0.001	0.013	0.001	0.011
CHOP (fold)	0.83 ± 0.09	0.66 ± 0.08	0.45 ± 0.10	0.001	0.011	0.001	0.010
Serum GRP78 (ng/mL)	32.55 ± 12.49	55.36 ± 14.78	78.14 ± 10.83	0.001	0.001	0.001	0.001
Serum CHOP (ng/mL)	66.94 ± 13.58	40.07 ± 9.92	20.50 ± 5.85	0.001	0.001	0.001	0.001

p; According to the “Kruskal Wallis” test and pairwise group comparisons were made according to the “Mann-Whitney U” test with Bonferroni adjustment for three comparisons.

Data are presented as mean ± SD.

P values <0.05 were considered statistically significant.

CHOP, C/EBP homologous protein; GRP78, glucose-regulated protein 78; miR, microRNA; PCa, prostate cancer.

Using the miRWalk database, several target genes of miR-188-5p were identified, including GRP78 and CHOP. The scores used in the analyses are binding probability scores generated by the machine-learning (Random Forest)- based TarPmiR algorithm, representing the probability that a candidate region is the “true target site”. In this scoring system, significant variations in the estimated binding strength were observed depending on the location of the targeted mRNA region (3’UTR or 5’UTR). As a result of the in-silico analyses performed, the binding score for the 3’UTR region of the DDIT3 (CHOP) gene was determined as 0.85. Specifically for the HSPA5 (GRP78) gene, a score of 0.85 was obtained for the 3’UTR region, while the interaction probability in the 5’UTR region was calculated as 1.

An analysis of the binding sites between miR-188-5p and its target genes revealed distinct interaction patterns. For the CHOP gene, no complementary pairing was identified within the canonical 3′ seed region. However, evaluation of non-seed (3′ seedless) regions demonstrated that, for binding sites with a 3′ bp value of 0, the calculated ΔG was −29, whereas for those with a 3′ bp value of 1, the ΔG was −20. These findings suggest that CHOP interaction predominantly occurs in non-seed regions (**Figure 1A**).

**Figure 1:**
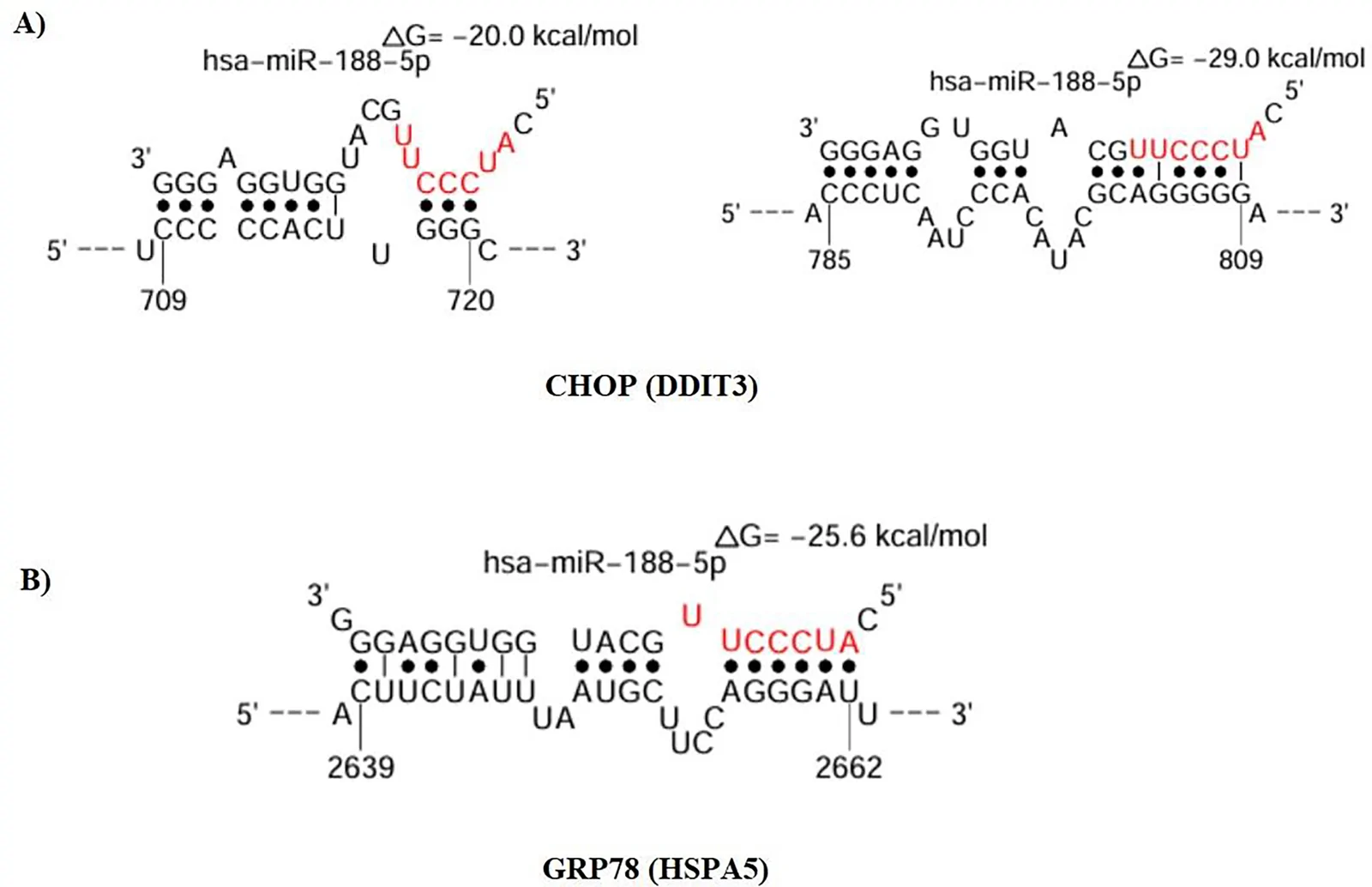
**(A)** Interaction of miR-188-5p with DDIT3 in seedless regions. **(B)** Interaction of miR-188-5p with HSPA5 in seed regions.

In contrast, analysis of the GRP78 gene indicated a canonical 3′ seed match, with a corresponding ΔG value of −25.6, although the 3′_bp value for this interaction was 0. Further assessment of seedless interactions revealed that when the 3′ base pairing (3′_bp) was 1, the ΔG value was −25.1, whereas a 3′_bp value of 0 corresponded to a ΔG of −27.6. Collectively, these results indicate that both seed-mediated and non-seed interactions contribute to miR-188-5p binding to GRP78, with varying binding energies (**Figure 1B**).

The differential expression patterns of GRP78 and CHOP in PRAD were investigated using the GEPIA3 database. After integration with normal tissue data obtained from the TCGA and GTEx repositories, GRP78 expression levels were found to be significantly elevated in PRAD tissues compared with normal controls, whereas CHOP expression was significantly reduced (p < 0.05) (**Figure 2**).

**Figure 2:**
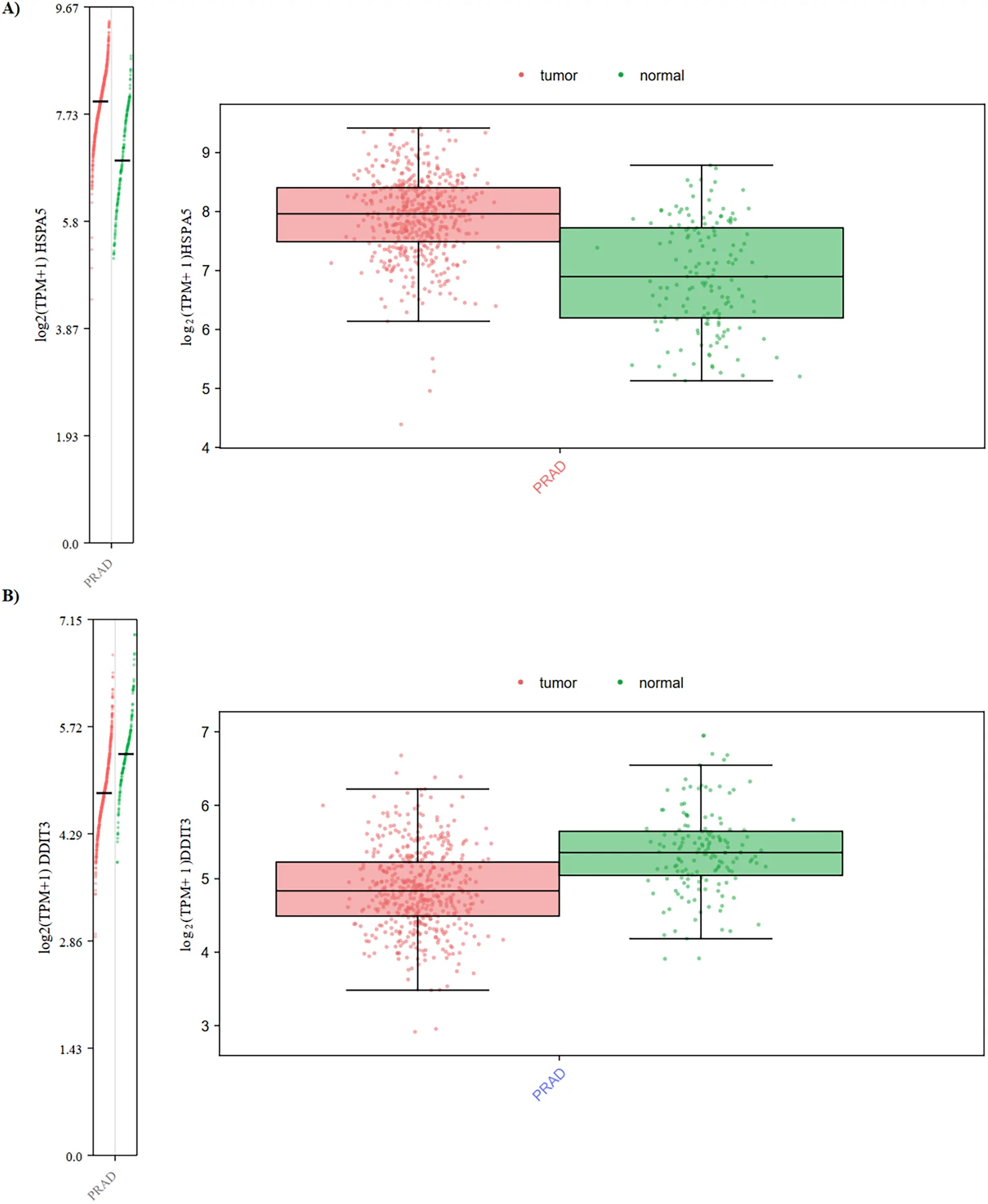
**(A)** GRP78 expression is shown to be upregulated (median_tumor = 7.96, median_normal= 6.90 and p-val= 1.41e-40) and **(B)** CHOP downregulated (median_tumor = 4.84, median_normal= 5.36 and p-val= 2.14e-21) in PRAD compared with normal tissues, based on TCGA normal and GTEx data analyzed through the GEPIA3 database (N tumor=496, N normal=152).

Correlation analysis was performed to explore the relationships among the molecular biomarkers. A strong positive association was observed between miR-188-5p and GRP78, whereas miR-188-5p showed an inverse correlation with CHOP. Similarly, GRP78 was negatively correlated with CHOP. The corresponding correlation matrix is presented in **Figure 3**.

**Figure 3:**
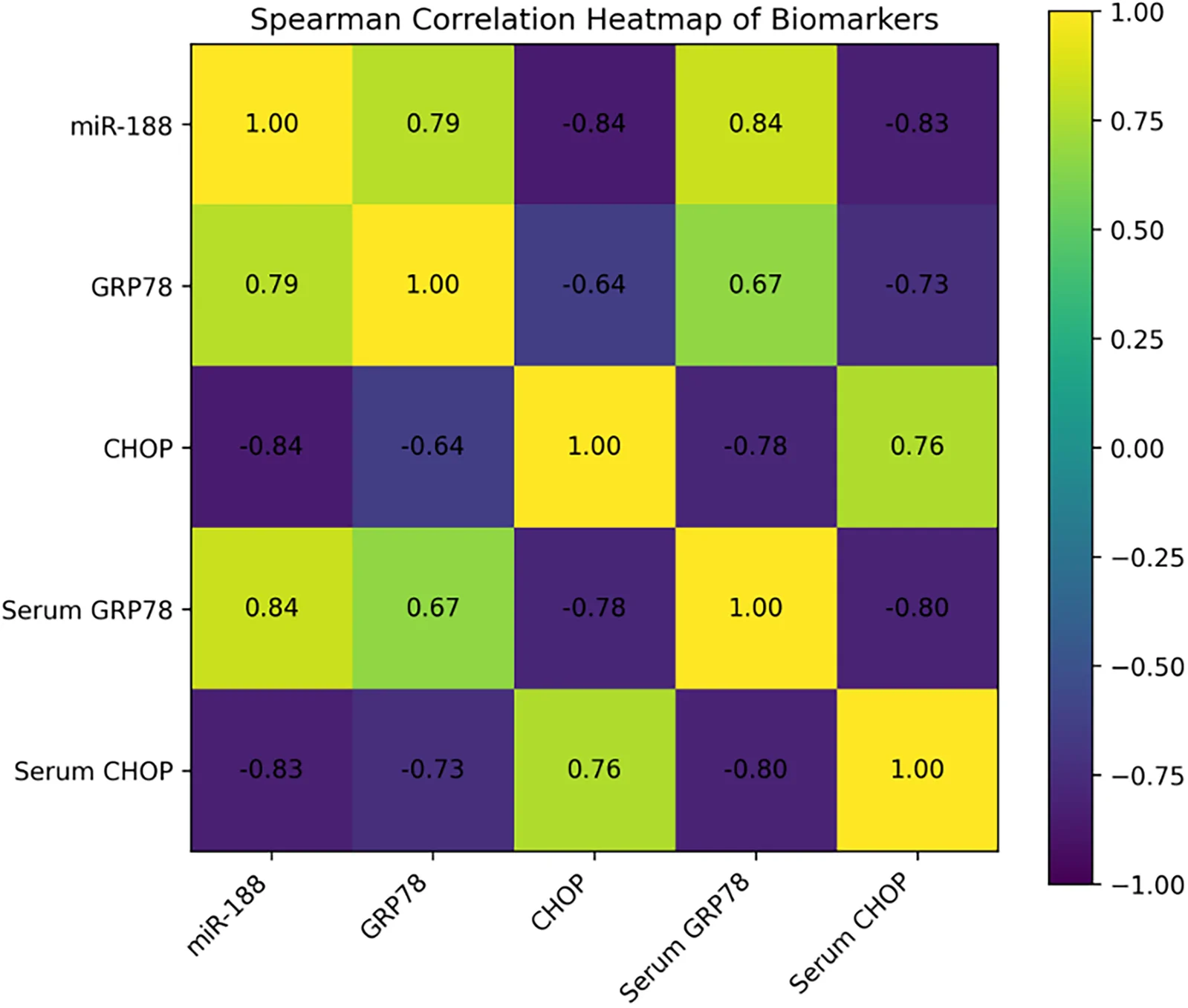
Heatmap showing correlations between biomarkers.

ROC curve indicated that tissue-based biomarkers displayed robust diagnostic efficacy in differentiating prostate cancer patients from controls. Among these, miR-188-5p exhibited the strongest discriminative capacity, followed by GRP78 and CHOP expression levels. Each of the three markers has excellent sensitivity and specificity on its own, which means they could be useful as diagnostic indicators. Notably, when these biomarkers were combined into a composite model, the diagnostic performance improved substantially, reaching an area under the curve (AUC) of 0.966, which reflects excellent discriminatory power. This finding indicates that the incorporation of miR-188-5p with ER stress-related indicators improves diagnostic precision compared to single-marker methodologies. The better results of the combined model show how useful it is to look at both regulatory miRNAs and ER stress components in the same biological setting (**Figure 4A**).

**Figure 4:**
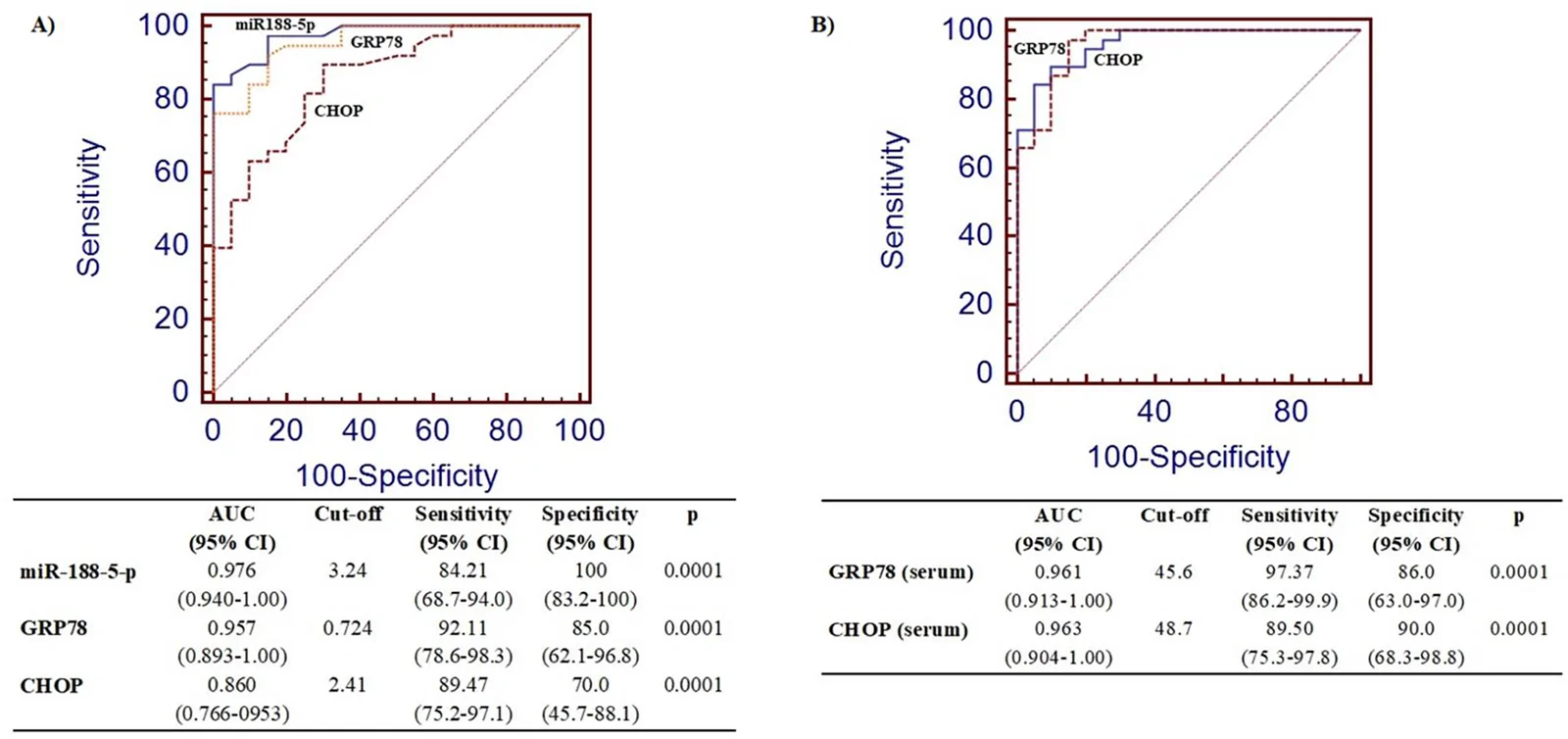
ROC curves of the study groups. **(A)** ROC curve for miR-188-5p, GRP78 and CHOP expression levels; **(B)** ROC curve for serum GRP78 and CHOP levels in the study group.

ROC curve analysis of serum biomarkers exhibited considerable diagnostic potential. Serum GRP78 had significant discriminative capability, while serum CHOP displayed an opposite yet diagnostically pertinent pattern, aligning with its biological function as a pro-apoptotic marker. While blood biomarkers exhibited slightly lower diagnostic performance relative to tissue-based markers, they nonetheless attained clinically significant AUC values, suggesting their viability as non-invasive tools for diagnosis. Significantly, the contrasting directional changes noted between serum GRP78 (elevated) and serum CHOP (reduced) reinforce the notion of a dysregulated ER stress response in prostate cancer. This complementary pattern may improve diagnostic interpretation when both indicators are assessed concurrently (**Figure 4B**).

The relationship between biopsy-determined tumor aggressiveness and biomarker levels was evaluated using plots. The analysis demonstrated that as the degree of biopsy proven aggressiveness increased, the levels of miR-188-5p and GRP78 increased progressively. In contrast, CHOP expression, both in tissue and serum, exhibited a decreasing trend with increasing aggressiveness. The levels of the studied biomarkers in relation to patients’ biopsy results are illustrated in **Figure 5**.

**Figure 5:**
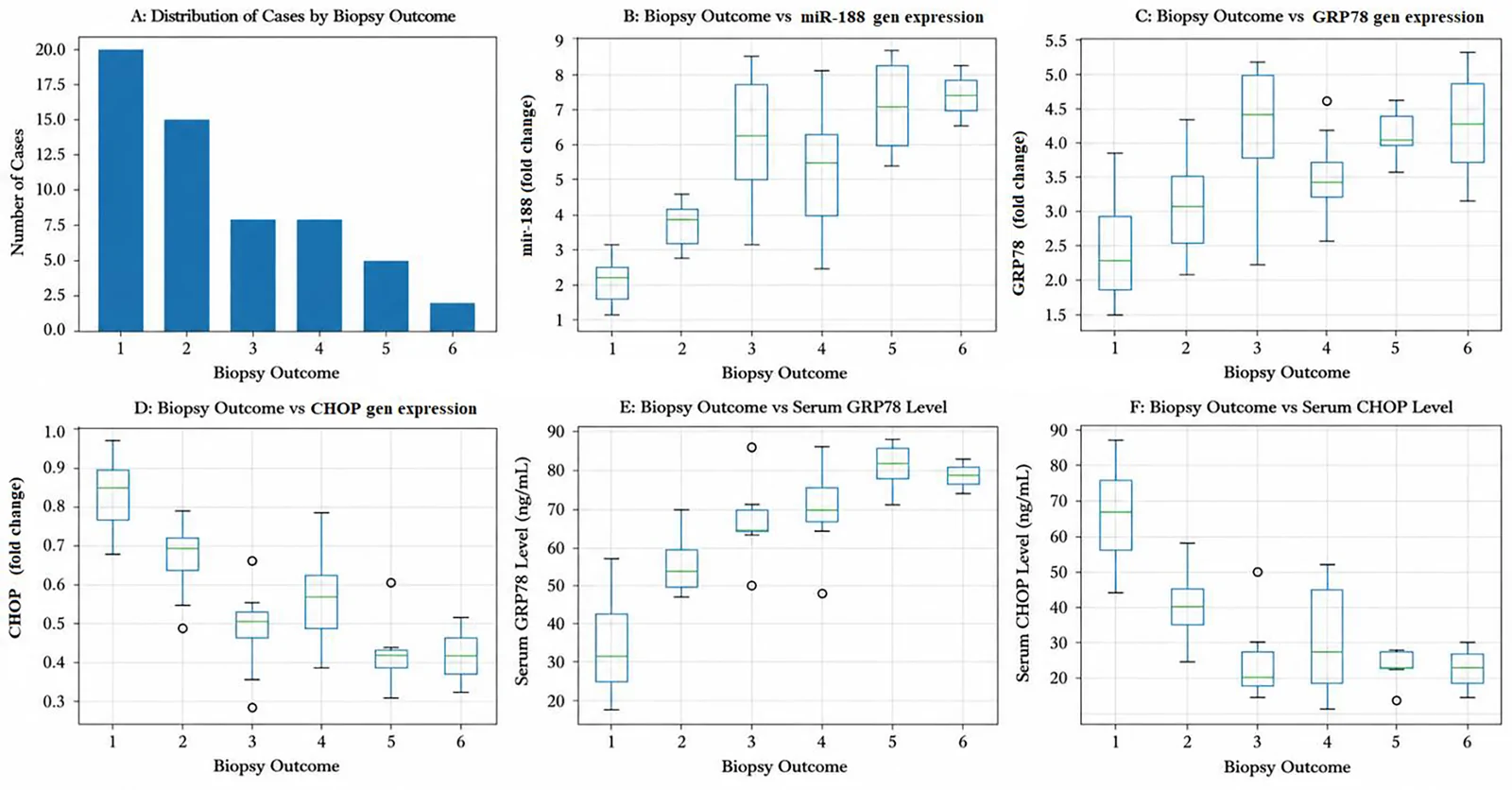
Distribution of biomarker levels according to biopsy-defined disease aggressiveness. **(A)** Distribution of participants across biopsy categories. **(B)** miR-188-5p expression. **(C)** Tissue GRP78 expression. **(D)** Tissue CHOP expression. **(E)** Serum GRP78 concentration. **(F)** Serum CHOP concentration. Biopsy categories were coded as follows: 1, benign or normal prostate tissue (n = 20); 2, Gleason score 6 (3 + 3; n = 15); 3, Gleason score 7 (3 + 4; n = 8); 4, Gleason score 7 (4 + 3; n = 8); 5, Gleason score 8 (4 + 4; n = 5); and 6, Gleason score ≥9 (4 + 5, 5 + 4, or 5 + 5; n = 2). In panels B–F, the center line represents the median, the box represents the interquartile range, the whiskers extend to 1.5 times the interquartile range, and circles indicate potential outliers. These panels provide a descriptive visualization of biomarker distributions across biopsy categories; no inferential statistical tests or significance symbols are presented in this figure. Total sample size was n = 58.

Exploratory univariate logistic regression showed statistically significant associations between cancer presence and PSA, PSAD, miR-188-5p, tissue GRP78 and CHOP, and serum GRP78 and CHOP (**Table 3**). The previously multivariable model was removed because the miR-188-5p estimate was highly unstable (OR = 403.02; 95% CI: 6.07–26,718.42), indicating substantial overfitting in this small cohort. Accordingly, the present results do not establish independent predictive value, and age remains a potential confounder.

**Table 3:** Exploratory univariate logistic regression for cancer presence.

	β	Odds ratio	%95 CI	p-value
PSA	0.130	1.14	1.02–1.28	**0.024**
PSAD	1.964	3.31	1.32–8.26	**0.010**
miR-188-5p	4.291	73.06	4.15–1287.74	**0.003**
GRP78 (tissue)	1.841	6.30	2.37–16.73	**0.001**
CHOP (tissue)	-25.751	6.5516 × 10^−12^	2.3535 × 10^−18^ – 1.8238 × 10^−5^	**0.001**
Serum GRP78	0.221	1.25	1.08–1.44	**0.002**
Serum CHOP	-0.225	0.80	0.69–0.92	**0.002**

Bold p-values indicate statistical significance (p < 0.05).

A cribriform pattern was observed among the 14 patients diagnosed with prostate cancer. Univariate logistic regression analysis demonstrated that elevated levels of miR-188-5p and serum GRP78 were significant positive predictors for the presence of cribriform morphology. Conversely, higher tissue and serum CHOP levels were significantly associated with a decreased likelihood of exhibiting a cribriform pattern (**Table 4**).

**Table 4:** Exploratory univariate logistic regression for the presence of cribriform patterns.

	β	Odds Ratio	%95 CI	p-value
PSA	0.033	1.03	0.99–1.08	**0.132**
PSAD	1.326	3.77	0.76–18.59	**0.103**
miR-188-5p	0.459	1.58	1.08–2.31	**0.018**
GRP78 (tissue)	0.380	1.46	0.70–3.06	**0.314**
CHOP (tissue)	-7.939	2.8254 × 10^−4^	6.0373 × 10^−7^ – 0.16	**0.010**
Serum GRP78	0.102	1.11	1.04–1.18	**<0.001**
Serum CHOP	-0.126	0.88	0.81–0.95	**0.001**

Bold p-values indicate statistical significance (p < 0.05).

Differential expression analysis revealed that miR-188-5p, tissue GRP78, and serum GRP78 levels were significantly elevated in the prostate cancer group compared with the control group. In contrast, tissue CHOP and serum CHOP levels were significantly decreased (all FDR < 0.05). Among the biomarkers examined, the greatest change in expression was observed for miR-188-5p (log2FC = 1.28), suggesting a strong association between miR-188-5p expression and the presence of prostate cancer (**Table 5**).

**Table 5:** Differential expression in this study.

	Cancer mean	Control mean	log2FC	p value
miR-188-5p	5.34	2.19	1.28	**<0.001**
GRP78 (tissue)	3.56	2.30	0.63	**<0.001**
CHOP (tissue)	0.56	0.83	−0.56	**<0.001**
GRP78 (serum)	66.48	32.55	1.03	**<0.001**
CHOP (serum)	31.26	66.94	−1.10	**<0.001**

Bold p-values indicate statistical significance (p < 0.05).

## 4 Discussion

Prostate carcinoma is one of the most common malignancies among men worldwide and represents a leading cause of cancer-related mortality (13). Despite advancements in diagnostic techniques and multimodal therapeutic strategies, including surgery, radiotherapy, and immunotherapy (14–16), clinical outcomes for patients with advanced-stage PCa remain suboptimal. In this situation, finding new molecular biomarkers that show how tumors work beyond standard clinical characteristics is still a very important necessity in the treatment of prostate cancer (17).

Factors such as ER stress and periprostatic adipose tissue can influence the prostate tumor microenvironment and may affect tumor progression and therapeutic response. The present study contributes clinical expression data showing that ER stress-related markers are altered in prostate cancer and associated with biopsy-defined aggressiveness. These associations do not establish that miR-188-5p directly regulates the GRP78–CHOP pathway or identify a therapeutic target (18).

GRP78/BiP, a key regulator of the ER stress response, is well recognized for its critical role in cancer biology. Similar to observations in many solid tumors, GRP78 expression is elevated in prostate cancer and has been reported to be associated with tumor progression (19). In line with these data, our results demonstrated a progressive elevation in GRP78 expression alongside a corresponding reduction in CHOP levels as tumor grade increased, corroborating the concept that tumor cells choose adaptive ER stress signaling over apoptosis. In contrast, CHOP, a protein involved in the pro-apoptotic arm of the UPR, is generally linked to the induction of cellular apoptosis under prolonged ER stress. However, recent studies have indicated that CHOP expression in cancer cells may exert context-dependent effects. Some reports suggest that reduced CHOP expression can facilitate tumor cell adaptation to stressful conditions (20).

miRNAs can directly target oncogenes or tumor suppressor genes, thereby playing critical roles in carcinogenesis and tumor progression (21, 22). Consequently, miRNAs represent strategic candidates for early-stage screening, diagnostic evaluation, and therapeutic intervention (23, 24). Although the clinical utility of dysregulated miRNA expression profiles for tumor classification and prognostic assessment is well established, miRNA dysregulation is a defining feature during the development of PCa. Nevertheless, the molecular mechanisms underlying miRNA-associated expression changes in PCa remain incompletely understood. In particular, the relationship between miR-188-5p and ER stress-related markers warrants further investigation. In this study, we aimed to investigate the expression profiles of GRP78, CHOP, and miR-188-5p in prostate cancer biopsy samples across different risk categories, elucidate their clinicopathological associations, and explore predicted target genes and interaction networks related to ER stress.

miR-188-5p expression was significantly elevated in PCa tissue samples compared with controls. Previous studies have described context-dependent tumor-suppressive or cancer-associated roles for miR-188-5p in several malignancies, including prostate cancer (9, 25, 26). Differences among reports may reflect disease stage, model system, cellular heterogeneity, normalization strategy, or sample size. In this cohort, increasing miR-188-5p expression was associated with biopsy-defined aggressiveness; however, the observational design does not establish an oncogenic function or therapeutic target status. Larger, independent cohorts and functional experiments are required.

Bioinformatic analyses predicted possible miR-188-5p binding sites in HSPA5 and DDIT3 transcripts. These predictions, together with the observed expression correlations, are hypothesis-generating only. They may reflect direct repression, indirect regulation, compensatory feedback, shared upstream signaling, or changes in the cellular composition of biopsy tissue. In particular, the positive correlation between miR-188-5p and GRP78 is not the pattern expected for a simple direct repressive interaction. Therefore, HSPA5 and DDIT3 are described as candidate predicted targets, and the findings should not be interpreted as evidence of a confirmed regulatory axis.

Analysis of GRP78 and CHOP expression in PCa revealed that GRP78 levels are elevated in both tissue and serum. In contrast, CHOP, a pro-apoptotic transcription factor, is generally suppressed or remains at low levels. This pattern may facilitate cancer cell adaptation to stress conditions, thereby promoting cell survival. These findings are consistent with the results obtained from bioinformatic analyses. Moreover, similar observations regarding GRP78 and CHOP expression have been reported in previous studies on prostate cancer (19, 27–30).

Tissue-level protein expression was not measured by immunohistochemistry or Western blotting. Consequently, the serum ELISA findings cannot establish intracellular accumulation, cellular localization, or cell-surface redistribution of GRP78 within prostate tissue, nor can serum protein concentrations be treated as direct validation of tissue mRNA expression. Tissue-based protein assays are required in future studies to determine whether the observed transcript changes correspond to localized protein alterations.

miR-188-5p was positively correlated with GRP78 and inversely correlated with CHOP, while GRP78 was negatively correlated with CHOP. Because miRNAs generally repress their direct targets, the concordant increase in miR-188-5p and GRP78 argues against a simple direct repressive relationship. The pattern may instead reflect tumor-grade effects, common upstream ER-stress signaling, compensatory feedback, indirect regulation, or cellular heterogeneity. Thus, these data indicate coordinated expression changes rather than a molecular switch or a demonstrated regulatory mechanism.

The positive correlation between miR-188-5p and GRP78 requires cautious interpretation because miRNAs typically reduce the expression of directly targeted transcripts through mRNA degradation or repression. Therefore, the observed positive correlation does not provide evidence that miR-188-5p directly induces GRP78 expression. Because both miR-188-5p and GRP78 increased with tumor aggressiveness, their positive association may be partly attributable to their shared relationship with disease grade or ER stress intensity. Alternatively, the pattern may reflect indirect regulatory pathways, compensatory feedback mechanisms, or differences in the cellular composition of bulk biopsy specimens. Furthermore, a miRNA and its predicted target can show concordant expression across heterogeneous clinical samples when both are influenced by a common upstream factor, even if the miRNA exerts an inhibitory effect within individual cells. Consequently, the present correlation should be regarded as an expression-level association rather than evidence of direct or positive regulation.

The increased expression of miR-188-5p observed in the high-grade PCa group appears to contrast with previous reports describing miR-188-5p as a tumor-suppressive miRNA. However, this finding should not necessarily be interpreted as evidence that miR-188-5p acquires a direct oncogenic function in advanced PCa. The expression and biological effects of miRNAs are highly context-dependent and may vary according to tumor stage, cellular composition, tumor microenvironment, molecular subtype, and the availability of target transcripts. Therefore, the increase observed in our cohort may represent a stage-associated alteration or a compensatory cellular response to progressive tumor-related and ER stress rather than a causal driver of tumor aggressiveness. Differences between our findings and those of previous studies may also be related to clinical and methodological heterogeneity. Variations in Gleason score and ISUP grade distribution, disease stage, treatment status, sample source and processing, normalization strategy, and patient-related characteristics may influence the reported miRNA expression profiles. Moreover, ethnic and geographical differences may affect both tumor molecular characteristics and miRNA expression; however, the present study was not designed or sufficiently powered to assess ethnicity-specific effects. Accordingly, our results indicate an association between increased miR-188-5p expression and aggressive clinicopathological features in this particular cohort but do not demonstrate that miR-188-5p promotes prostate cancer progression. Larger multicenter studies involving clinically and ethnically diverse populations are required to determine whether the observed expression pattern is reproducible and genuinely stage-specific.

The tissue and serum ER stress markers and miR-188-5p were associated with PCa presence and biopsy-defined aggressiveness in this cohort. The parallel increase in miR-188-5p and GRP78 should be interpreted as an expression-level association and not as evidence that miR-188-5p directly activates GRP78 or drives PCa progression. The ROC findings suggest that combined marker assessment may warrant further investigation. However, all models were developed and evaluated in the same small cohort without internal resampling or external validation; consequently, the reported discrimination may be optimistic and should not be interpreted as established clinical utility.

Serum GRP78 increased and serum CHOP decreased across the study groups. These circulating patterns may be relevant to tumor-associated ER stress, but serum measurements do not validate tissue protein abundance, intracellular localization, or cell-surface relocation. Their potential as non-invasive biomarkers requires confirmation in larger, independent cohorts.

Exploratory univariate regression demonstrated associations between miR-188-5p and PCa presence and between several markers and cribriform morphology. Because of the limited sample size, model instability, and absence of age-adjusted validation, these findings should not be interpreted as independent predictive effects.

The cribriform pattern is widely recognized as a high-risk histopathological feature linked to aggressive tumor behavior and unfavorable clinical outcomes in PCa. Recent evidence indicates that this architectural pattern may also be associated with an increased risk of biochemical recurrence and metastasis (31). In this context, the observed relationship among miR-188-5p, ER stress–related markers, and cribriform morphology suggests that these molecular alterations may be closely linked to tumor aggressiveness and disease progression. These findings suggest that ER stress-related molecular alterations may not only reflect tumor presence but also provide insight into histopathological features associated with aggressive disease behavior.

This study has several limitations. First, direct binding of miR-188-5p to HSPA5 or DDIT3 was not tested using dual-luciferase assays with wild-type and mutant constructs, and gain- or loss-of-function experiments with miR-188-5p mimics or inhibitors were not performed. The study therefore cannot establish direct regulation or causality. Second, GRP78 and CHOP proteins were measured in serum but not in prostate tissue by immunohistochemistry or Western blotting, precluding conclusions about tissue abundance or localization. Third, the cohort was small and single-center. The initially fitted PSA + miR-188-5p multivariable model yielded an extremely wide confidence interval, indicating overfitting; it was therefore removed, and only exploratory univariate estimates are presented. Age differed between controls and high-risk cases, but a stable age-adjusted model could not be supported, so residual confounding is possible. Finally, ROC and composite-model performance were assessed without independent validation and may be optimistic. Future multicenter studies should incorporate larger age-balanced cohorts, tissue-level protein validation, luciferase assays, miRNA perturbation experiments, and penalized regression with internal and external validation. Nevertheless, this study provides clinically grounded, paired tissue-transcript and serum-protein data on miR-188-5p, GRP78, and CHOP across biopsy-defined PCa risk groups. The results identify reproducible expression-level associations and generate testable hypotheses for future mechanistic and biomarker-validation studies.

## 5 Conclusion

Stage-associated changes in miR-188-5p, GRP78, and CHOP were associated with prostate cancer presence and biopsy-defined aggressiveness in this cohort. Computational predictions and clinical correlations suggest a potential relationship between miR-188-5p and ER stress-related markers but do not demonstrate direct target binding, regulation, or causality. The diagnostic estimates are exploratory and require confirmation in larger, age-adjusted independent cohorts with tissue-level protein assessment and functional validation.

## Data Availability

The public datasets analyzed in this study are available through TCGA and GTEx and were accessed using GEPIA3, as described in the article. De-identified clinical and experimental data are available from the corresponding authors upon reasonable request, subject to applicable ethical and institutional requirements.
